# Flooding is a key driver of the Tonle Sap dai fishery in Cambodia

**DOI:** 10.1038/s41598-021-81248-x

**Published:** 2021-02-15

**Authors:** Ashley S. Halls, Kent G. Hortle

**Affiliations:** 1 Aquae Sulis Research Ltd, Midway House, Turleigh, Wiltshire BA15 2LR UK; 2 Institute of Land, Water and Society, Charles Sturt University, PO Box 789, Albury, NSW 2640 Australia

**Keywords:** Freshwater ecology, Environmental impact, Hydrology

## Introduction

**arising from**: P. B. Ngor et al.; *Scientific Reports* 10.1038/s41598-018-27340-1 (2018).

As one of the richest sources of fisheries-related data in the lower Mekong basin, the Tonle Sap dai fishery has received considerable attention in the literature in recent years as concerns grow over the impacts of hydropower dams on fisheries, which are important for livelihoods and food security^1–3^.

Ngor et al.^4^ reported a decline since 2000 in the catch of larger species which tend to occupy higher trophic levels; compensatory increases in the catch of smaller species; and declines in the mean body weight (and length) of common species in the Tonle Sap dai fishery, as evidence of the effects of indiscriminate fishing or “fishing-down” of the multi-species fish assemblage in the lower Mekong basin. We provide evidence below that suggest that these apparent recent changes are more likely to reflect changing hydrological conditions than fishing-down effects, possibly caused by climate change and recently also by hydropower development.

**Figure 1:**
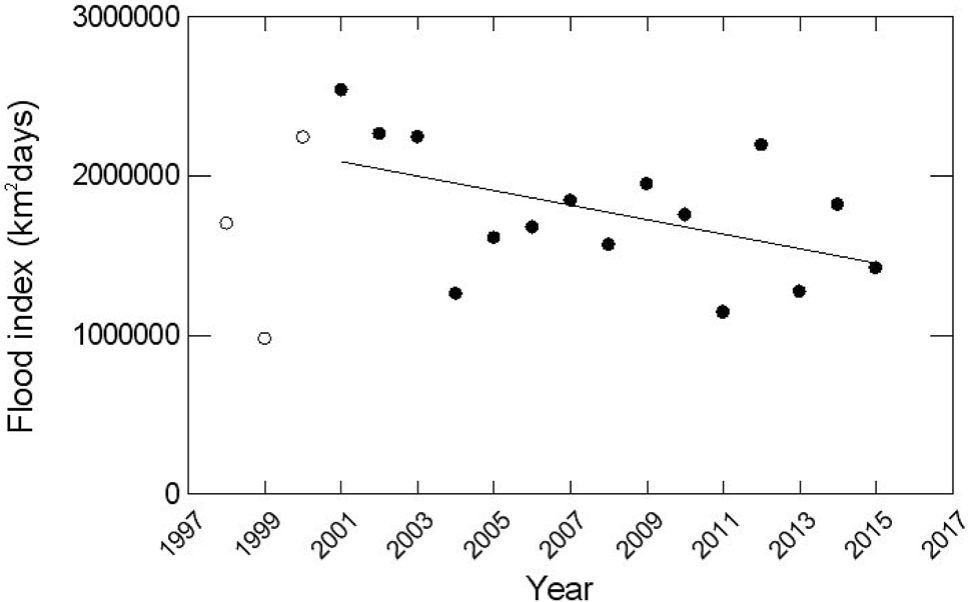
The flood index (FI) or flood pulse^14^ in the Tonle Sap Great Lake System (1997/08–2014/15). The FI is a measure of the flood extent and duration, calculated as the sum of the flooded area days above the mean flooded area from April to March of the following year^2^. Whilst highly variable, a downward decline (*p*-value = 0.06) in the FI is observed between 2000/01 (Year 2001) and 2014/15 (Year 2015) shown by solid circles. Adding the most recent data for 2016–2018 (not shown here), confirmed that a downward linear trend in the FI since the 2000/01 season is statistically significant (*p*-value < 0.01). Data Source: Mekong River Commission Secretariat.

The dai fishery has been reliably monitored since 1997–98. Without explanation, Ngor et al. excluded the first three seasons (1997–98 to 1999–2000) of monitoring data which include one of the driest fishing seasons on record (1998–99). The authors thereby created a time series beginning with the three wettest seasons (largest floods) since monitoring began (2000–1 to 2002–3) that were followed by 12 seasons of variable, but decreasing flows caused by hydropower dam construction, low rainfalls possibly resulting from climate change, and abstractions for agriculture^5,6^ (Fig. 1).

Without reference to hydrological variation, Ngor et al. reported a temporal decline in the catch of larger, high-trophic level species; compensatory increases in the catch of smaller species; and declines in the mean body weight (and length) of six common species. The authors reported these findings as evidence of the effects of indiscriminate fishing (fishing-down) in the lower Mekong basin, without considering alternative explanations for the observed patterns.

Ngor et al. describe their dataset as being generated from a “standardized biological catch assessment”. In reality, this assessment is complex having undergone numerous changes through time, including to sampling effort^2^. Moreover, the fishery itself is not standardised, with net types, mesh sizes and flow rates through the gears varying inter and intra-annually. Catches may therefore not reflect changes in underlying abundance^7^. Despite this, we re-examined the analysis of Ngor et al. at face value.

Using the 15-season dataset that accompanied Ngor et al., we re-fitted regression models to the log_e_-transformed catch time series of larger species (TL > 45 cm) excluding those with zero catch in any year. These 28 species formed approximately 16% of the total catch during the study period. We also found negative regression coefficients for all 28 species, supporting the findings of Ngor et al. However, the combined annual catch of these 28 species did not decline significantly through time (R^2^ = 0.22; *p*-value = 0.07).

**Figure 2:**
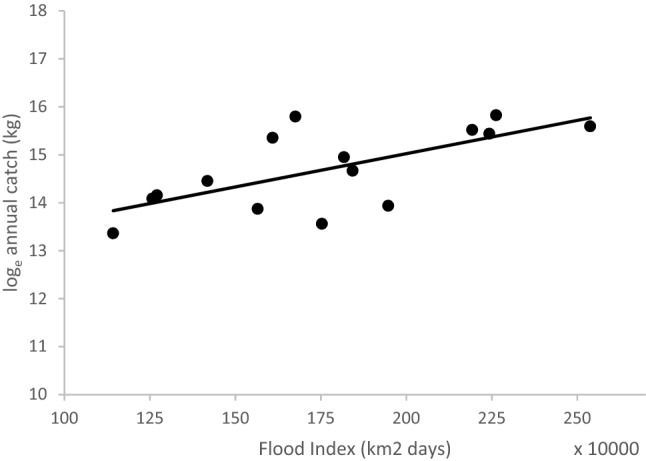
The relationship between the log_e_-transformed annual catch of larger (TL > 45 cm) species and the flood index (R^2^ = 0.46; *p*-value < 0.01).

We did however find that the combined annual catch of these 28 larger species varied significantly with the annual flood index (FI)—a measure of flood extent and duration (R^2^ = 0.46; *p*-value < 0.01) (Fig. 2). The FI is a key driver of fish growth and fisheries productivity in floodplain-river systems^8^.

Annual catches for each of the 28 species were also found to vary (correlate positively) with the FI. R^2^ values also improved for 23 of the 28 regression models when ‘year’ was replaced by FI as the independent explanatory variable (Table 1). The five species for which R^2^ values did not improve formed less than 1% of the catch reported for the study period.

**Table 1:** Comparison of R^2^ values for regression models of log_e_ annual catch (dependent variable) of larger species with either ‘year’ or the ‘flood index’ as the explanatory variable.

Species	Max. TL (cm)	Trophic level	Catch (kg) (2001–15)	% of total catch 116 species (2001–15)	Regression model R^2^	
					Year (fishing effort)	Flood index
All Spp > 45 cm	49.8–240	2–4.08	49,472,406	16.3%	0.22 (*p*-value = 0.07)	0.46 (*p*-value < 0.01)
*Amblyrhynchichthys truncatus*	48.8	2.4	2,784,494	0.9%	0.20	0.59
*Arius maculatus*	80	3.36	38,180	0.0%	0.03	0.17
*Belodontichthys truncatus*	73.2	4.08	3,738,435	1.2%	0.15	0.23
*Boesemania microlepis*	122	3.72	1,242,872	0.4%	0.01	0.24
*Catlocarpio siamensis*	300	2.92	125,872	0.0%	0.05	0.27
*Channa striata*	122	3.36	70,330	0.0%	0.11	0.02
*Chitala ornata*	122	3.68	412,581	0.1%	0.29	0.21
*Cirrhinus microlepis*	79.3	2.38	6,136,799	2.0%	0.15	0.41
*Cosmochilus harmandi*	100	2	2,083,962	0.7%	0.00	0.23
*Cyclocheilichthys enoplos*	90.3	3.15	4,605,586	1.5%	0.39	0.58
*Helicophagus waandersii*	70	3.15	773,046	0.3%	0.55	0.56
*Hemibagrus nemurus*	79.3	3.62	1,666,100	0.5%	0.09	0.24
*Hemisilurus mekongensis*	80	3.3	108,837	0.0%	0.23	0.49
*Hypsibarbus malcolmi*	61	3.2	2,277,388	0.8%	0.23	0.27
*Labeo chrysophekadion*	90	2	7,881,750	2.6%	0.02	0.32
*Leptobarbus hoevenii*	122	2.76	412,659	0.1%	0.07	0.22
*Macrochirichthys macrochirus*	100	3.7	487,208	0.2%	0.32	0.53
*Notopterus notopterus*	73.2	3.6	141,934	0.0%	0.10	0.09
*Osteochilus melanopleurus*	73.2	2.32	2,268,628	0.7%	0.62	0.34
*Osteochilus schlegeli*	49	2	117,595	0.0%	0.00	0.08
*Oxyeleotris marmorata*	79.3	3.9	29,389	0.0%	0.02	0.29
*Pangasianodon hypophthalmus*	158.6	3.12	1,374,944	0.5%	0.28	0.55
*Pangasius bocourti*	146.4	3.18	123,503	0.0%	0.03	0.02
*Pangasius conchophilus*	146.4	2.73	1,563,026	0.5%	0.03	0.20
*Pangasius larnaudii*	158.6	3.26	4,593,059	1.5%	0.07	0.34
*Phalacronotus micronemus*	61	4.03	3,232,839	1.1%	0.20	0.27
*Probarbus jullieni*	183	3.17	877,647	0.3%	0.09	0.23
*Wallago attu*	240	3.68	303,743	0.1%	0.28	0.29
				Mean	0.16	0.30

Contrary to Ngor et al. we found no clear evidence of a compensatory response by small species. The annual catch of the three most prolific small species of the genus *Henicorhynchus*, that formed 42% of the total catch during the study period, exhibited no significant trend through time (Adjusted R^2^ =  − 0.0216, *p*-value = 0.42).

The growth of species of fish caught by the dai fishery is non-linear and seasonal^2^. The time series analysis of mean fish weight illustrated in Figure 4 of Ngor et al. was subject to sampling-related bias because the number of observations of fish weight in each month varied significantly each year.

To avoid this bias, and minimise any gear selectivity effects, we examined how the mean body weight of the six common species examined by Ngor et al. varied in both December and January each year corresponding to the end of the flood, when any flood-related effects on fish growth would manifest. We compared regressions of mean log_e_-transformed body weight using ‘year’ and the FI as alternative independent explanatory variables. To aid model comparisons we first standardised both independent variables.

The FI explained more of the variation in mean fish weight than ‘year’ in 9 of the 12 regressions compared (Table 2). Residual plots indicated heteroskedasticity in most cases, but for all six species, the flood index provided a much better explanatory model of the variation in mean fish body weight compared with ‘year’. Mean fish weight did not decline linearly during the 15-year study period but rather exhibited periods of increase and decrease in response to the FI.

**Table 2:** Comparison of R^2^ and coefficient values of regression models of mean log_e_ fish body weight (dependent variable) of the six common species of fish examined by Ngor et al. with either standardised ‘year’ or the ‘flood index’ as the explanatory variable.

Species	Month	Independent variable: standardised year			Independent variable: standardised flood index		
		R^2^	*b*	Residuals indicate	R^2^	*b*	Residuals indicate
*C. microlepis*	December	0.38	− 0.72	1	0.32	0.62	3
	January	0.13	− 0.28	1	0.18	0.32	5
*C. enoplos*	December	0.07	− 0.39	1	0.37	0.80	4
	January	0.11	− 0.49	3	0.23	0.64	5
*H. lobatus*	December	0.23	− 0.24	1	0.30	0.25	4
	January	0.27	− 0.19	2	0.26	0.17	5
*L. lineata*	December	0.11	− 0.16	3	0.22	0.22	5
	January	0.18	− 0.19	3	0.27	0.21	5
*O. melanopleurus*	December	0.18	− 0.76	3	0.26	0.85	5
	January	0.16	− 0.61	3	0.12	0.50	5
*P. hypophthalmus*	December	0.15	− 0.48	3	0.25	0.56	5
	January	0.07	− 0.30	3	0.12	0.37	5
Mean		0.17	− 0.40		0.24	0.46	

All regressions were significant (*p*-value < 0.001). Residuals Indicate: 1-Poor model fit; 2-Heteroskedasticity; 3-Heteroskedasticity and poor model fit; 4-Acceptable model fit; 5-Heteroskedasticity but acceptable model fit.

The FI also had a stronger effect on mean fish weight than ‘year’ as indicated by the regression coefficient values. These regression coefficients also indicate that the larger species are more sensitive to the effects of the flood index compared to the smaller species examined.

## Conclusions

Statistically significant relationships between the mean size of fish caught in the dai fishery and the Tonle Sap-Great Lake flood index have been previously reported in the literature^2,9–11^. Longer and more extensive flooding increases the availability of spawning and feeding habitat, and extends the seasonal growth period of fish. These and other studies including^1^ have presented evidence that fish biomass and catch increase in response to the flood index.

These growth and catch responses are also evident in the dataset accompanying Ngor et al., and appear most evident for larger species.

Whilst the growth of small, single-cohort, r-selected species responds to the flood index, order-of magnitude variations in recruitment to their populations probably mask these growth effects on their catches^9^.

In contrast, a legacy of poor growth during dry years is likely to persist for larger, longer-lived, later-maturing, K-selected species whose populations comprise multiple cohorts which are limited by habitat or resource availability, in this case the inundated floodplain. Sustained periods of declining flood-pulses are likely to have significant short-term and cumulative effects on mean body size, fecundity, survival, and ultimately catches of these large species.

There are no reliable estimates nor time series of fishing effort or mortality to support the claim that “fishing-down” is responsible for the apparent recent trends reported by Ngor et al. Indeed, fishing effort may have actually declined in Cambodia’s inland waters, following the removal of highly efficient industrial fishing operations (fishing lots) in Cambodia in 2012^12^, and as former fishers take-up alternative employment in booming regional economies.

While fishing pressure undoubtedly caused some fishing-down between 1970 and the 1990s corresponding to strong population growth and the spread of modern fishing methods^13^, we suspect that since then, an already fished-down assemblage has been further impacted by periods of low flow and limited flooding, possibly caused by climate change and hydropower dams^6^.

## References

[1] Sabo JL (2017). Designing river flows to improve food security futures in the Lower Mekong Basin. Science.

[2] Halls AS (2013). The stationary Trawl (Dai) fishery of the tonle sap-great lake, Cambodia. MRC Tech. Paper.

[3] Ziv G, Baran E, So N, Rodríguez-Iturbe I, Levin SA (2012). Trading-off fish biodiversity, food security, and hydropower in the Mekong River Basin. Proc. Natl. Acad. Sci..

[4] Ngor P (2018). Evidence of indiscriminate fishing effects in one of the world’s largest inland fisheries. Sci. Rep..

[5] Räsänen TA (2017). Observed river discharge changes due to hydropower operations in the Upper Mekong Basin. J. Hydrol..

[6] Pokhrel Y (2018). A review of the integrated effects of changing climate, land use, and dams on Mekong River hydrology. Water.

[7] Hayes, D. P., Ferreri, C. P. & Taylor, W. W. Active fish capture methods. in *Fisheries Techniques*, 3rd edn. (eds Zale, A. V. *et al.*) Ch. 7, 267-304 (American Fisheries Society, 2012).

[8] Welcomme RL (1985). River fisheries. FAO Fish. Tech. Paper.

[9] Halls AS (2013). Integrated analysis of data from MRC fisheries monitoring programmes in the Lower Mekong Basin. MRC Tech. Paper.

[10] Halls AS, Sopha L, Ngor P, Tun P (2008). New research reveals ecological insights into dai fishery. Catch Cult..

[11] Halls AS, Paxton BR (2012). The stationary Trawl (Dai) fishery of the tonle Sap-Great Lake system, Cambodia in Inland fisheries evolution and management: case studies from four continents. FAO Fish. Aquacult. Tech. Paper.

[12] Anon (2012). Cambodia abolishes fishing lots. Catch Cult..

[13] Hortle, K. G. Fisheries of the Mekong River Basin. in *The Mekong: Biophysical Environment of an International River Basin* (ed Campbell, I. C.) Ch. 9, 197-249 (Elsevier Publishers, 2009).

[14] Junk, W. J., Bayley, P. B. & Sparks, R. E. The flood pulse concept in river-floodplain systems. *Can. J. Fish. Aquat. Sci.* **106**, 110-127 (1989).

